# Interleukin-3 Polymorphism is Associated with Miscarriage of Fresh in Vitro Fertilization Cycles

**DOI:** 10.3390/ijerph16060995

**Published:** 2019-03-19

**Authors:** Cheng-Hsuan Wu, Tsung-Hsien Lee, Shun-Fa Yang, Hui-Mei Tsao, Yu-Jun Chang, Chia-Hsuan Chou, Maw-Sheng Lee

**Affiliations:** 1 Institute of Medicine, Chung Shan Medical University, Taichung 40201, Taiwan; 97528@cch.org.tw (C.-H.W.); ysf@csmu.edu.tw (S.-F.Y.); cwlin@csmu.edu.tw (C.-H.C.); 2 Women’s Health Research Laboratory, Changhua Christian Hospital, Changhua 50006, Taiwan; 3 School of Medicine, Kaohsiung Medical University, Kaohsiung 80708, Taiwan; 4 Department of Obstetrics and Gynecology, Chung Shan Medical University Hospital, Taichung 40201, Taiwan; 5 Department of Medical Research, Chung Shan Medical University Hospital, Taichung 40201, Taiwan; 6 Division of Infertility Clinic, Lee Womens’ Hospital, Taichung 406, Taiwan; cshn076@csh.org.tw; 7 Epidemiology and Biostatistics Center, Changhua Christian Hospital, Changhua 50006, Taiwan; 83686@cch.org.tw

**Keywords:** abortion, IVF outcomes, interleukin, polymorphism

## Abstract

The aim of this study was to examine the association between interleukin (IL) genes polymorphisms and in vitro fertilization (IVF) outcome. A prospective cohort analysis was performed at a Women’s Hospital IVF centre of 1015 female patients undergoing fresh non-donor IVF cycles. The effects of the following six single nucleotide polymorphisms (SNPs) in five IL genes on IVF outcomes were explored: IL-1α (rs1800587 C/T), IL-3 (rs40401 C/T), IL-6 (rs1800795 C/G), IL-15 (rs3806798 A/T), IL-18 (rs187238 C/G) and IL-18 (rs1946518 G/T). The main outcome measures included clinical pregnancy, embryo implantation, abortion and live birth rates. There were no statistically significant differences in clinical pregnancy, embryo implantation and live birth rates in the analysis of 1015 patients attempting their first cycle of IVF. Infertile women with IL-3 homozygous major genotype had a higher abortion rate than those with heterozygous and homozygous minor genotype (16.5% vs. 7.9%, P = 0.025). In conclusion, our results indicated that the IL-3 rs40401 polymorphism is associated with increased risk of abortion of IVF patients. Future studies with inclusion of other ethnic populations must be conducted to confirm the findings of this study.

## 1. Introduction

Despite numerous technical advances in the administration of in vitro fertilization (IVF) to infertile couples, embryo implantation remains the rate-limiting step for a successful IVF procedure [1]. The endometrium is receptive to the embryo only during a period known as the implantation window. During this period, both the developing embryo and the receptive endometrium undergo a synchronized complex process involving several factors including immune cells, growth factors, cytokines and adhesion molecules [2,3]. It is believed that the events underlying the endometrial receptivity are products of several genes; therefore, alterations due to polymorphisms in genes involved in embryo implantation could lead to IVF failure [4,5].

Among the large number of embryo implantation failure or miscarriage-related factors, interleukins those involved in abnormal immune reactions are particularly notable [6]. Interleukins are a group of immunomodulatory proteins that mediate several immune reactions in human body. The interleukin (IL)-1 system is composed of a family of peptides, including two agonists, IL-1α and IL-1β. IL-1α may have a critical function in the development of obesity [7]. Increasing body weight is associated with significantly lower progesterone concentrations in early pregnancy after blastocyst transfer and affects the IVF outcome [8]. IL-3 is a haematopoiesis promoting factor and aids in embryo implantation and placental development, whose maternal serum level increases as a function of the trimester [9]. IL-3 gene polymorphisms were associated with acute rejection after kidney transplantation [10]. IL-6 plays a role in reproduction and immune balance, acting as a multifunctional cytokine with anti- and pro-inflammatory response and regulates the behaviour of the gestational tissues [11]. Liu et al. demonstrated a possible association between IL-6-174 promoter polymorphism and recurrent spontaneous abortion [12]. IL-15 is implicated in the recruitment and proliferation of uterine natural killer (NK) cells in the endometrium [13]. Increased expression of IL-15 in the placenta tissue of patients may be connected to disturbed implantation with following foetal loss [14]. IL-18, mainly produced by macrophages, stimulates IFN-γ production and activates NK and T cells, which are involved in uterine implantation [15]. Serum IL-18 level is associated with response to ovarian stimulation and plays an important role in a successful pregnancy outcome after IVF treatment [16].

Studies have illustrated that genetic variations in IL genes responsible for regulating the activity of the immune system might cause embryo implantation failure or early pregnancy loss [17]. After meta-analysis, a systematic review indicated that a significant association between IL-1β (-511C/T) polymorphism and recurrent miscarriage (RM) [18]. Likewise, research conducted in Iranian women has revealed a relationship between the *IL-6* rs1800796 (−634C/G) polymorphism and an increased risk of RM [19]. In addition, Yue et al. showed that the IL-18-137G/C gene polymorphism may contribute to pathogenesis of idiopathic RM in Chinese Han population. Al-Khateeb et al. reported that two SNPs of IL-18 (rs360717 and rs1946519) are significantly associated with RM [15]. Finally, the effects of IL-3 and IL-15 polymorphisms on pregnancy outcomes have not been studied yet. It must be highlighted that none of the above studies were conducted in an IVF population. Therefore, we decided to assess the possible correlations between the IL polymorphisms and pregnancy outcomes based on IVF population. Our findings would be of value as a prognostic indicator of successful IVF outcomes.

## 2. Materials and Methods

### 2.1. Study Design and Subjects

Women undergoing IVF at the Lee Women’s Hospital in Taichung, Taiwan (n = 1015) were prospectively enrolled in the study from January 2014 to December 2015. The causes of infertility included male factor, tubal factor, endometriosis, polycystic ovarian syndrome (PCOS), advance maternal age, unexplained and others. Eligibility inclusion criteria were: (1) a woman age ≤ 40 years old; (2) undergoing the first IVF or intracytoplasmic sperm injection (ICSI) cycle; and (3) blastocyst transfer in the fresh cycle. Exclusion criteria were autoimmune dysfunction, genetic anomalies, inflammatory disease and other systemic disorders. A venous blood sample was drawn for DNA extraction with subsequent interleukin genotyping. Ethics approval (CS13194) was obtained from the Institutional Review Board of Chung Shan Medical University Hospital. 

We studied the effects of the following six single-nucleotide polymorphisms (SNPs) in five IL genes on IVF outcomes: IL-1α C/T rs1800587 (C___9546481_20), IL-3 C/T rs40401 (C___2397269_30), IL-6 C/G rs1800795 (C___1839697_20), IL-15 A/T rs3806798 (C__27519194_10), IL-18 C/G rs187238 (C___2408543_10) and IL-18 G/T rs1946518 (C___2898460_10). The details of the selected SNP were described in supplementary Table S1. Relevant IVF outcomes were clinical pregnancy, embryo implantation, abortion and live birth. Clinical pregnancy was defined as the presence of an intrauterine gestational sac (GS). Embryo implantation rate was calculated by dividing the number of implanted GS by the number of embryos transferred. Spontaneous abortion is defined as the natural loss of a pregnancy with an ultrasound-confirmed GS before 20 gestational weeks. Live birth was considered birth after 24 weeks of gestation.

### 2.2. IVF Treatment Protocol

The details of a stimulation cycle procedure have been previously described [20]. Patients underwent either a standard long protocol using the gonadotrophin-releasing hormone (GnRH) agonist or a short protocol using GnRH antagonists. The long protocol began with daily subcutaneous injections of 0.5 mg of leuprolide acetate (Lupron; Takeda Pharmaceutics, Konstantz, Germany) from cycle Day 21 of the previous cycle. On cycle day 3, recombinant FSH (Gonal-F, Merck-Serono, Darmstadt, Germany) or highly purified FSH (Menopur; Ferring Pharmaceuticals, Kiel, Germany) was administered via individual set with flexible doses. Patients on the antagonist protocol had gonadotrophin stimulation initiated on day 3 of the cycle and the GnRH antagonist (Orgalutran; Organon Laboratories) at a daily dose of 0.25 mg was administered using a fixed day 6 protocol. Final oocyte maturation was triggered with 10,000 IU human Chorionic Gonadotropin (Profasi; Serono) and oocyte retrieval was performed 36 to 38 hours later. Fertilization was carried out either by conventional insemination or ICSI depending on the semen parameters. Fresh blastocyst transfer was performed throughout the study period.

### 2.3. DNA Extraction and Determination of Genotypes

Genomic DNA was extracted from EDTA anti-coagulated venous blood using a QIAamp DNA blood mini kit (Qiagen, Valencia, USA), according to the manufacturer’s instructions described in detail previously [21]. DNA was dissolved in TE buffer (10 mM Tris and 1 mM EDTA acid; pH 7.8) and then quantitated by a measurement of the optical density at 260 nm. The final preparation was stored at −20 °C and used as templates for polymerase chain reaction (PCR). Allelic discrimination of the six studied SNPs was assessed with the ABI StepOne™ Real-Time PCR System (Applied Biosystems, Foster City, CA, USA) and analysed using SDS vers. 3.0 software (Applied Biosystems, Foster City, CA, USA), with the TaqMan assay [22].

### 2.4. Statistical Analysis

The demographic data and other clinically relevant data of continuous variables are presented as mean and standard deviation, whereas categorical variables were presented as percentage. The chi-square test was used to investigate the different effects of IL polymorphisms on IVF outcome. Continuous variables between the 2 groups were compared using the Student’s t-test. All data were analysed using the IBM SPSS Statistics for Windows, Version 22.0 (IBM Corp., Armonk, NY). *p*-values < 0.05 were considered statistically significant.

## 3. Results

There were 1015 patients attempting their first fresh cycle of IVF. The genotypic frequencies of IL SNPs are shown in Table 1. Genotypic distributions of IL-1α (rs1800587 C/T), IL-3 (rs40401 C/T), IL-18 (rs187238 C/G) and IL-18 (rs1946518 G/T) conformed to Hardy-Weinberg equilibrium (*p* = 0.469, χ2 value: 0.525; *p* = 0.486, χ2 value: 0.528; *p* = 0.330, χ2 value: 0.950 and *p* = 0.873, χ2 value: 0.025; respectively), except for IL-15 (rs3806798 A/T) (*p* = 0.030, χ2 value: 4.723). The following notation will be used for describing the genotypes of all SNPs: homozygous major as “AA,” heterozygous as “Aa” and homozygous minor as “aa.” The genotypes frequencies of IL-6 polymorphism were AA (100%), Aa (0%) and aa (0%). Table 2 outlines the baseline and IVF treatment cycle characteristics of 1015 IVF patients.

Table 3 shows the association between IL polymorphisms and IVF outcomes. There were no statistically significant differences found in clinical pregnancy, embryo implantation, abortion or live birth rates. We did not examine the association between IL-6 polymorphism and IVF outcomes because of the 100% frequency of the homozygous major genotype. Combining the genotypes with the minor allele (Aa/aa) and comparing them to the homozygous major genotype (AA) yielded statistically insignificant results for clinical pregnancy, embryo implantation or live birth rates. However, a statistically significant difference in abortion rate was found for the recessive model of IL-3 SNP (16.5% for AA and 7.9% for Aa/aa, *p* = 0.025) (Table 4).

The baseline and IVF treatment cycle characteristics of IL-3 polymorphism patients were shown in Table 5. The baseline characteristics of the both subgroups (AA and Aa/aa), especially age, ovarian reserve indexes and infertility duration, were not statistically different. Also, no significant differences were observed in the factors thought to affect the outcome measures during IVF treatment, including the number of mature oocytes, fertilization rates, Day 5 good embryo rate, number of transferred embryos and endometrial thickness.

## 4. Discussion

Throughout its 40-year history, IVF has improved significantly. However, implantation and pregnancy rates with IVF have increased slowly year after year [23]. Therefore, a more specific recognition of patients’ individual genetic profiles may be helpful in the appraisal of each woman’s intrinsic capacity to become pregnant. In this study, the maternal genetic profile at six gene polymorphisms associated with immune and inflammation were investigated in a cohort of IVF patients and IL-3 was shown to be associated with IVF abortion. To the best of our knowledge, our study is the first to report that the IL-3 genetic polymorphism can affect abortion rates in Taiwan Han IVF population.

It must be emphasized that only one study was done to investigate the relationships between the IL polymorphisms and IVF outcomes and found that IL-1 receptor antagonist gene polymorphism is associated with a poor prognosis of obtaining a pregnancy after IVF [24]. In our study, we found no association between IL-1α, IL-15 or IL-18 SNPs and IVF outcomes. However, a meta-analysis showed that the IL-18 polymorphism is associated with an increased risk of recurrent pregnancy loss [18]. A possible explanation for this discrepancy is that our study was conducted in an IVF population, whereas the populations pooled for the meta-analysis were not IVF subjects.

This study revealed that *IL-3* rs40401 C/T polymorphisms had higher abortion rate in IVF patients. Spontaneous miscarriage is the most common complication of pregnancy and occurs in 10–15% of pregnancies during the first trimester [25]. The mechanisms of pregnancy loss are not completely understood. It is believed that apart from chromosomal, embryonic and anatomical defects, immunologic rejection of the embryo by the maternal immune system may be involved [26].

To maintain a healthy pregnancy, human endometrial endothelial cells (HEECs) produce a number of chemokines and proangiogenic factors that may promote immune cell recruitment, trophoblast invasion and spiral artery remodelling, which establishes the adequate placentation and vascular development [27]. Women with antiphospholipid antibodies (aPL) are at risk for pregnancy loss [28]. Previous in vitro studies have revealed that aPL inhibits HEEC vascular endothelial growth factor secretion and their ability to form vessel-like structures [29,30]. A systemic review also showed that aPL can directly affect trophoblast function and lead to impaired placentation and vascular transformation [31].

Women with pregnancy loss due to the anti-phospholipid antibody syndrome (APS) have reduced levels of IL-3 [32]. IL-3 belongs to the family of hematopoietic cytokines [33] and is a growth factor for the trophoblast that assists in embryo implantation and placental development [34]. Mice with aPL-induced foetal loss have low levels of IL-3 and administration of recombinant IL-3 to the affected mice increased pregnancy success [35]. The mechanism by which IL-3 reverses the effects of aPL on pregnancy is not completely understood. Di Simone et al. [36] submitted that IL-3 can reverse the effects of aPL on hormone secretion and trophoblast invasion. However, Chamley et al. question if it has the same effects on human trophoblasts [37]. Savion et al. showed the ability of ciprofloxacin to reduce pregnancy loss in the mouse model, possibly through elevation of IL-3 [38]. 

Furthermore, IL-3 is the main cytokine for the generation of dendritic cells (DCs), which play a role in inducing T helper 2 (Th2) cell responses [39]. IL-3-treated monocyte-derived DC shifts Th cell responses toward a Th2 cytokine pattern [39]. Thus, the activity of IL-3 may influence the balance between Th1 and Th2 cytokines. Imbalance in Th1/Th2 cytokine level is a critical factor associated with foetus abortion. Th1 type response has been related to pregnancy failure, whereas the Th2 type dominant response has been associated with normal pregnancy [40]. Significantly elevated level of Th1/Th2 cytokine is present in infertile women with multiple implantation failures after IVF and in women with recurrent pregnancy losses [41]. 

Maternal age has great impact on IVF pregnancy outcome. The association of IL SNPs with pregnancy outcome was further analysed by stratification with age of 35 in Table 3 and Table 4. We found that the impact of IL-3 SNP on abortion is borderline significantly for young age patients, which suggest that the effect of IL-3 SNP on abortion is not associated with advanced maternal age.

This study has multiple strengths. First, it used a large prospective sample size, which decreases the risk for selection bias. Second, this study included all patients undergoing their first fresh IVF cycle and consented to DNA use. Including patients who failed multiple cycles will obscure any effects of the SNPs because other factors, such as poor oocyte quality, are more likely to significantly influence the IVF outcomes. Third, we collected live birth data, which is ultimately the outcome of interest to the patient.

## 5. Conclusions

In summary, our results indicated that IL-3 variant is associated with increased risk of abortion of IVF patients. The limitation of the study was performed only in an IVF population of Taiwan Han ethnicity. Future more large-scale population studies with inclusion of other ethnic backgrounds must be conducted to confirm that IL-3 variant rs40401 is associated with IVF abortion. The mechanism underlying the role of IL-3 in abortion remains to be determined. To define the role of IL-3, comprehensive functional experiments are also needed.

## Figures and Tables

**Table 1:** The frequencies of IL-1α, IL-3, IL-6, IL-15 and IL-18 polymorphisms in in vitro fertilization (IVF) women.

Variable	AA		Aa		aa		A Allele		a Allele	
*n* = 1015	N	%	N	%	N	%	N	%	N	%
IL-1α C/T	884	87.1	128	12.6	3	0.3	1896	93.3	134	6.6
IL-3 C/T	253	24.9	519	51.1	243	23.9	1025	50.4	1005	49.5
IL-6 C/G	1015	100.0	0	0.0	0	0.0	2030	100	0	0
IL-15 A/T	739	72.8	264	26.0	12	1.2	1742	85.8	288	14.1
IL-18 C/G	748	73.7	251	24.7	16	1.6	1747	86	283	13.9
IL-18 G/T	257	25.3	510	50.2	248	24.4	1024	50.4	1006	49.5

AA: homozygous major, Aa: heterozygous, aa: homozygous minor.

**Table 2:** Clinical characteristics of women undergoing IVF treatment.

Number of Patients	1015
Mean patient age (years)	35.5 ± 4.6
Cause of infertility	
Male factor (%)	208 (20.5%)
Tubal factor (%)	196 (19.3%)
Endometriosis (%)	73 (7.2%)
PCOS (%)	97 (9.6%)
Unexplained (%)	79 (7.8%)
Advance maternal age (%)	273 (26.9%)
Multiple factors (%)	89 (8.7%)
Total gonadotropins administered (IU)	2921 ± 892
Basal FSH (mIU/mL)	7.1 ± 4.4
Basal LH (mIU/mL)	6.5 ± 0.3
Basal AMH (mIU/mL)	3.1 ± 0.1
Basal estradiol (pg/mL)	52.3 ± 2.5
Peak estradiol (pg/mL) *	1892 ± 1342
No. of oocytes retrieved	10 ± 6.6
No. of blastocyst stage embryos	4.8 ±2.4
No. of embryos transferred	2.5 ± 0.9
Fertilization rate (%)	82.6 ± 21.1
Endometrial thickness (mm) **	11.8 ± 2.1

Values presented as mean ± standard deviation. * levels on the trigger day, ** thickness on the day of embryo transfer.

**Table 3:** Effect of Interleukin polymorphisms on IVF outcome.

Variable		Age≤35				Age>35				Overall			
		AA	Aa	aa	*p*-Value	AA	Aa	aa	*p*-Value	AA	Aa	aa	*p*-Value
**Clinical pregnancy**													
IL-1α	N	180/444	28/64	2/2	0.242	100/440	15/64	0/1	0.903	280/884	43/128	2/3	0.358
	%	40.5	43.8	100.0		22.7	23.4	0.0		31.7	33.6	66.7	
	OR	1.000	1.141	-		1.000	1.041	0.000		1.000	1.091	4.314	
IL-3	N	55/117	94/250	61/143	0.213	30/136	61/269	24/100	0.939	85/253	155/519	85/243	0.305
	%	47.0	37.6	42.7		22.1	22.7	24.0		33.6	29.9	35.0	
	OR	1.000	0.679	0.839		1.000	1.036	1.116		1.000	0.842	1.063	
IL-15	N	154/378	56/130	0/2	0.550	78/361	35/134	2/10	0.555	232/739	91/264	2/12	0.340
	%	40.7	43.1	0.0		21.6	26.1	20.0		31.4	34.5	16.7	
	OR	1.000	1.101	0.000		1.000	1.283	0.907		1.000	1.150	0.437	
IL-18 C/G	N	156/372	51/130	3/8	0.871	83/376	29/121	3/8	0.552	239/748	80/251	6/16	0.894
	%	41.9	39.2	37.5		22.1	24.0	37.5		32.0	31.9	37.5	
	OR	1.000	0.894	0.831		1.000	1.113	2.118		1.000	0.996	1.278	
IL-18 G/T	N	54/124	100/258	56/128	0.533	32/133	54/252	29/120	0.772	86/257	154/510	85/248	0.448
	%	43.5	38.8	43.8		24.1	21.4	24.2		33.5	30.2	34.3	
	OR	1.000	0.820	1.008		1.000	0.861	1.006		1.000	0.860	1.037	
**Implantation**													
IL-1α	N	254/1070	40/151	3/5	0.122	133/1142	18/161	0/2	1.000	387/2212	58/312	3/7	0.195
	%	23.7	26.5	60.0		11.6	11.2	0.0		17.5	18.6	42.9	
	OR	1.000	1.158	4.819		1.000	0.955	0.000		1.000	1.077	3.537	
IL-3	N	78/285	131/600	88/341	0.145	42/360	81/702	28/243	0.998	120/645	212/1302	116/584	0.133
	%	27.4	21.8	25.8		11.7	11.5	11.5		18.6	16.3	19.9	
	OR	1.000	0.741	0.923		1.000	0.988	0.986		1.000	0.851	1.084	
IL-15	N	216/900	81/321	0/5	0.406	107/930	41/346	3/29	0.964	323/1830	122/667	3/34	0.368
	%	24.0	25.2	0.0		11.5	11.8	10.3		17.7	18.3	8.8	
	OR	1.000	1.069	0.000		1.000	1.034	0.887		1.000	1.044	0.452	
IL-18 C/G	N	219/896	75/311	3/19	0.683	108/982	39/302	4/21	0.369	327/1878	114/613	7/40	0.800
	%	24.4	24.1	15.8		11.0	12.9	19.0		17.4	18.6	17.5	
	OR	1.000	0.982	0.580		1.000	1.200	1.904		1.000	1.084	1.006	
IL-18 G/T	N	69/296	147/615	81/315	0.759	39/345	75/640	37/320	0.981	108/641	222/1255	118/635	0.719
	%	23.3	23.9	25.7		11.3	11.7	11.6		16.8	17.7	18.6	
	OR	1.000	1.033	1.139		1.000	1.042	1.026		1.000	1.061	1.126	
**Abortion**													
IL-1α	N	13/180	1/28	0/2	0.737	16/100	3/15		0.712	29/280	4/43	0/2	1.000
	%	7.2	3.6	0.0		16.0	20.0			10.4	9.3	0.0	
	OR	1.000	0.476	0.000		1.000	1.313			1.000	0.888	0.000	
IL-3	N	7/55	5/94	2/61	0.121	7/30	6/61	6/24	0.110	14/85	11/155	8/85	0.069
	%	12.7	5.3	3.3		23.3	9.8	25.0		16.5	7.1	9.4	
	OR	1.000	0.385	0.232		1.000	0.358	1.095		1.000	0.387	0.527	
IL-15	N	8/154	6/56		0.208	13/78	6/35	0/2	1.000	21/232	12/91	0/2	0.441
	%	5.2	10.7			16.7	17.1	0.0		9.1	13.2	0.0	
	OR	1.000	2.190			1.000	1.034	0.000		1.000	1.526	0.000	
IL-18 C/G	N	12/156	2/51	0/3	0.614	14/83	5/29	0/3	1.000	26/239	7/80	0/6	0.610
	%	7.7	3.9	0.0		16.9	17.2	0.0		10.9	8.8	0.0	
	OR	1.000	0.490	0.000		1.000	1.027	0.000		1.000	0.786	0.000	
IL-18 G/T	N	5/54	6/100	3/56	0.721	5/32	9/54	5/29	0.985	10/86	15/154	8/85	0.867
	%	9.3	6.0	5.4		15.6	16.7	17.2		11.6	9.7	9.4	
	OR	1.000	0.626	0.555		1.000	1.080	1.125		1.000	0.820	0.790	
**Live birth**													
IL-1α	N	159/444	27/64	2/2	0.098	73/440	9/64	0/1	0.764	232/884	36/128	2/3	0.234
	%	35.8	42.2	100.0		16.6	14.1	0.0		26.2	28.1	66.7	
	OR	1.000	1.308	-		1.000	0.823	0.000		1.000	1.100	5.621	
IL-3	N	45/117	85/250	58/143	0.397	19/136	46/269	17/100	0.703	64/253	131/519	75/243	0.226
	%	38.5	34.0	40.6		14.0	17.1	17.0		25.3	25.2	30.9	
	OR	1.000	0.824	1.092		1.000	1.270	1.261		1.000	0.997	1.318	
IL-15	N	138/378	50/130	0/2	0.650	58/361	22/134	2/10	0.944	196/739	72/264	2/12	0.715
	%	36.5	38.5	0.0		16.1	16.4	20.0		26.5	27.3	16.7	
	OR	1.000	1.087	0.000		1.000	1.026	1.306		1.000	1.039	0.554	
IL-18 C/G	N	140/372	45/130	3/8	0.828	60/376	20/121	2/8	0.786	200/748	65/251	5/16	0.883
	%	37.6	34.6	37.5		16.0	16.5	25.0		26.7	25.9	31.3	
	OR	1.000	0.877	0.994		1.000	1.043	1.756		1.000	0.958	1.245	
IL-18 G/T	N	46/124	92/258	50/128	0.807	19/133	40/252	23/120	0.562	65/257	132/510	73/248	0.501
	%	37.1	35.7	39.1		14.3	15.9	19.2		25.3	25.9	29.4	
	OR	1.000	0.940	1.087		1.000	1.132	1.423		1.000	1.032	1.232	

AA: homozygous major, Aa: heterozygous, aa: homozygous minor, OR: Odds ratio. *p*-Value by Chi-square test or Fisher’s exact test when appropriated.

**Table 4:** Effect of Interleukin polymorphisms on IVF outcome (recessive model).

Variable		Age≤35			Age>35			Overall		
		AA	Aa/aa	*p*-Value	AA	Aa/aa	*p*-Value	AA	Aa/aa	*p*-Value
**Clinical pregnancy**										
IL-1α	N	180/444	30/66	0.449	100/440	15/65	0.950	280/884	45/131	0.540
	%	40.5	45.5		22.7	23.1		31.7	34.4	
	OR	1.000	1.222		1.000	1.020		1.000	1.129	
IL-3	N	55/117	155/393	0.144	30/136	85/369	0.816	85/253	240/762	0.535
	%	47.0	39.4		22.1	23.0		33.6	31.5	
	OR	1.000	0.734		1.000	1.058		1.000	0.909	
IL-15	N	154/378	56/132	0.735	78/361	37/144	0.323	232/739	93/276	0.484
	%	40.7	42.4		21.6	25.7		31.4	33.7	
	OR	1.000	1.072		1.000	1.255		1.000	1.111	
IL-18 C/G	N	156/372	54/138	0.567	83/376	32/129	0.523	239/748	86/267	0.938
	%	41.9	39.1		22.1	24.8		32.0	32.2	
	OR	1.000	0.890		1.000	1.165		1.000	1.012	
IL-18 G/T	N	54/124	156/386	0.537	32/133	83/372	0.680	86/257	239/758	0.566
	%	43.5	40.4		24.1	22.3		33.5	31.5	
	OR	1.000	0.879		1.000	0.906		1.000	0.916	
**Implantation**										
IL-1α	N	254/1070	43/156	0.297	133/1142	18/163	0.822	387/2212	61/319	0.477
	%	23.7	27.6		11.6	11.0		17.5	19.1	
	OR	1.000	1.222		1.000	0.942		1.000	1.115	
IL-3	N	78/285	219/941	0.157	42/360	109/945	0.947	120/645	328/1886	0.486
	%	27.4	23.3		11.7	11.5		18.6	17.4	
	OR	1.000	0.805		1.000	0.987		1.000	0.921	
IL-15	N	216/900	81/326	0.760	107/930	44/375	0.907	323/1830	125/701	0.915
	%	24.0	24.8		11.5	11.7		17.7	17.8	
	OR	1.000	1.047		1.000	1.022		1.000	1.013	
IL-18 C/G	N	219/896	78/330	0.770	108/982	43/323	0.259	327/1878	121/653	0.519
	%	24.4	23.6		11.0	13.3		17.4	18.5	
	OR	1.000	0.957		1.000	1.243		1.000	1.079	
IL-18 G/T	N	69/296	228/930	0.673	39/345	112/960	0.857	108/641	340/1890	0.513
	%	23.3	24.5		11.3	11.7		16.8	18.0	
	OR	1.000	1.068		1.000	1.036		1.000	1.083	
**Abortion**										
IL-1α	N	13/180	1/30	0.698	16/100	3/15	0.712	29/280	4/45	1.000
	%	7.2%	3.3%		16.0%	20.0%		10.4%	8.9%	
	OR	1.000	0.443		1.000	1.313		1.000	0.844	
IL-3	N	7/55	7/155	0.055	7/30	12/85	0.261	14/85	19/240	0.025
	%	12.7	4.5		23.3	14.1		16.5	7.9	
	OR	1.000	0.324		1.000	0.540		1.000	0.436	
IL-15	N	8/154	6/56	0.208	13/78	6/37	0.952	21/232	12/93	0.299
	%	5.2	10.7		16.7	16.2		9.1	12.9	
	OR	1.000	2.190		1.000	0.968		1.000	1.489	
IL-18 C/G	N	12/156	2/54	0.527	14/83	5/32	0.872	26/239	7/86	0.471
	%	7.7	3.7		16.9	15.6		10.9	8.1	
	OR	1.000	0.462		1.000	0.913		1.000	0.726	
IL-18 G/T	N	5/54	9/156	0.358	5/32	14/83	0.872	10/86	23/239	0.598
	%	9.3	5.8		15.6	16.9		11.6	9.6	
	OR	1.000	0.600		1.000	1.096		1.000	0.809	
**Live birth**										
IL-1α	N	159/444	29/66	0.202	73/440	9/65	0.575	232/884	38/131	0.504
	%	35.8	43.9		16.6	13.8		26.2	29.0	
	OR	1.000	1.405		1.000	0.808		1.000	1.148	
IL-3	N	45/117	143/393	0.683	19/136	63/369	0.402	64/253	206/762	0.588
	%	38.5	36.4		14.0	17.1		25.3	27.0	
	OR	1.000	0.915		1.000	1.268		1.000	1.094	
IL-15	N	138/378	50/132	0.779	58/361	24/144	0.869	196/739	74/276	0.926
	%	36.5	37.9		16.1	16.7		26.5	26.8	
	OR	1.000	1.060		1.000	1.045		1.000	1.015	
IL-18 C/G	N	140/372	48/138	0.553	60/376	22/129	0.771	200/748	70/267	0.869
	%	37.6	34.8		16.0	17.1		26.7	26.2	
	OR	1.000	0.884		1.000	1.083		1.000	0.974	
IL-18 G/T	N	46/124	142/386	0.950	19/133	63/372	0.477	65/257	205/758	0.583
	%	37.1	36.8		14.3	16.9		25.3	27.0	
	OR	1.000	0.987		1.000	1.223		1.000	1.095	

AA: homozygous major, Aa: heterozygous, aa: homozygous minor, OR: Odds ratio. *p*-Value by Chi-square test or Fisher’s exact test when appropriated.

**Table 5:** Baseline characteristics and IVF treatment cycle parameters of IL-3 polymorphisms patients.

Variable	IL-3		
	CC (n = 253)	CT/TT (n = 762)	*p*-Value
Age (years)	35.81 ± 4.82	35.46 ± 4.48	0.308
Duration of infertility (years)	3.54 ± 2.84	3.72 ± 3.20	0.415
Total gonadotropin doses (IU)	2350.32 ± 1512.96	2283.76 ± 1405.19	0.622
Basal FSH (mIU/mL)	7.28 ± 5.04	6.94 ± 3.86	0.266
Basal LH (mIU/mL)	5.63 ± 4.59	5.75 ± 5.78	0.760
Basal AMH (mIU/mL)	3.11 ± 2.89	3.15 ± 2.92	0.834
Basal oestradiol (pg/mL)	36.48 ± 24.43	37.15 ± 25.14	0.711
Peak estradiol (pg/mL) *	1835.5 ± 1310.89	1910.04 ± 1351.89	0.509
P4 ng/ml at HCG injection day	2.01 ± 12.73	1.55 ± 6.79	0.526
No. of oocytes retrieved	9.52 ± 6.31	10.20 ± 6.73	0.162
Mature oocytes	7.54 ± 5.56	8.33 ± 6.36	0.080
Fertilization rate (%)	81.59 ± 22.41	82.99 ± 20.58	0.481
Day3 embryo no.	7.82 ± 5.41	7.57 ± 5.16	0.641
Day3 good embryo rate (%)	59.56 ± 22.67	61.63 ± 23.38	0.393
Day5 good embryo rate (%)	45.93 ± 21.24	47.11 ± 22.78	0.734
No. of transferred embryos	2.55 ± 0.90	2.48 ± 0.85	0.256
Endometrial thickness (mm) **	11.83 ± 1.86	11.78 ± 2.24	0.819
Clinical pregnancy (%)	85/253 (33.6%)	240/762 (31.5%)	0.535
Implantation (%)	120/645 (18.6%)	328/1886 (17.4%)	0.486
Abortion (%)	14/85 (16.5%)	19/240 (7.9%)	0.025
Abortion per cycle (%)	14/253 (5.5%)	19/762 (2.5%)	0.018
Live birth (%)	64/253 (25.3%)	206/762 (27.0%)	0.588
Aetiology of infertility			
Male factor (%)	53 (21.0)	174 (22.8)	0.286
Tubal factor (%)	44 (17.4)	137 (18.0)	0.507
Endometriosis (%)	15 (5.9)	47 (6.2)	1.000
PCOS (%)	25 (9.9)	81 (10.6)	0.745
Unexplained (%)	14 (5.5)	67 (8.8)	0.313
Advance maternal age (%)	78 (30.8)	197 (25.9)	0.744
Multiple factors (%)	24 (9.5)	59 (7.7)	1.000

CC: homozygous major, CT: heterozygous, TT: homozygous minor. Values presented as mean ± standard deviation; *p*-Value by Student’s t-test. * levels on the trigger day, ** thickness on the day of embryo transfer.

## Supplementary Materials

The following are available online at https://www.mdpi.com/1660-4601/16/6/995/s1, Table S1: Description of selected single-nucleotide polymorphism (SNP).
