# Supplementary material for: Interleukin-3 Polymorphism is Associated with Miscarriage of Fresh in Vitro Fertilization Cycles
Source: Int J Environ Res Public Health. 2019 Mar 19;16(6):995. doi: 10.3390/ijerph16060995 (PMC6466610; doi:10.3390/ijerph16060995)
Supplement: Supplementary file 1 [file ijerph-16-00995-s001.pdf]

Supplementary Table 1: Description of selected single-nucleotide polymorphism (SNP)

Genotype	SNP ID	Chromosome	Functional Consequence
IL-1α C/T	rs1800587	2	-949C>T; utr variant 5 prime
IL-3 C/T	rs40401	5	intron variant, missense
IL-6 C/G	rs1800795	7	-634C>G; intron variant, upstream variant 2KB
IL-15 A/T	rs3806798	4	-470T>A; upstream variant 2KB
IL-18 C/G	rs187238	11	-137G>C; upstream variant 2KB
IL-18 G/T	rs1946518	11	-838A>C; upstream variant 2KB
